# High Variability in Silver Particle Characteristics, Silver Concentrations, and Production Batches of Commercially Available Products Indicates the Need for a More Rigorous Approach

**DOI:** 10.3390/nano10071394

**Published:** 2020-07-17

**Authors:** Ilse De Leersnyder, Hannes Rijckaert, Leen De Gelder, Isabel Van Driessche, Pieter Vermeir

**Affiliations:** 1 Department of Green Chemistry and Technology, Laboratory of Chemical Analysis (LCA), Faculty of Bioscience Engineering, Ghent University, 9000 Ghent, Belgium; pieter.vermeir@ugent.be; 2 Department of Chemistry, Sol-gel Center for Research on Inorganic Powders and Thin film Synthesis (SCRiPTS), Faculty of Sciences, Ghent University, 9000 Ghent, Belgium; hannes.rijckaert@ugent.be (H.R.); Isabel.VanDriessche@ugent.be (I.V.D.); 3 Department of Biotechnology, Laboratory for Environmental Biotechnology, Faculty of Bioscience Engineering, Ghent University, 9000 Ghent, Belgium; leen.degelder@ugent.be

**Keywords:** consumer product, silver, colloidal, hydrosol, (nano)particles

## Abstract

Due to the beneficial properties of silver, it is anticipated that the number of commercially available applications will keep growing during the next decade. In this study, 14 different commercial products that claim to contain solid silver were characterized by visual analysis, UV-VIS spectroscopy, inductive coupled plasma optical emission spectrometry (ICP-OES), scanning transmission electron microscopy with energy dispersive x-ray spectroscopy (STEM-EDX), and dynamic light scattering (DLS). Moreover the variation between production batches—which has never been researched before—was investigated. All four techniques corroborated that some products were highly concentrated and contained spherically-shaped silver nanoparticles (AgNPs), while in others, no (solid) silver was detected or only irregularly-shaped silver particles with a high size polydispersity were present. For almost all products, a significant difference between the claimed and measured silver concentration was detected and a high variability between different production batches of the same product was observed. Our results show the need for a more rigorous approach regarding the manufacturing, labeling, and use of silver-containing products.

## 1. Introduction

Silver has been extensively used throughout history as a storage vessel for beverages and as a major therapeutic agent in medical formulations. Because of its antibacterial, antiviral, and antifungal properties, silver was the most important antimicrobial agent available before the introduction of antibiotics [1]. It has—up until now—been employed in a wide range of applications in various fields, such as health, cleaning, and food industries [2,3]. Some examples of its application are immune-supporting food supplements, wound dressings, burn treatment products, and disinfectant sprays [4,5,6,7]. To meet the diversity of application types, many different forms of silver compounds have been developed, such as silver salts and colloidal silver [8,9]. Moreover, the introduction of the term ‘silver nanoparticles (AgNPs)’ had a profound impact on silver usage and forested a new generation of commercial products with elemental silver [3,10]. Colloidal silver is a liquid dispersion of silver particles with a size range of 1–1000 nm [11,12,13], while AgNP suspensions are defined as having a range of 1–100 nm [14,15]. This means that formulations carrying the name colloidal silver applications could also be AgNP suspensions [8]. 

The antimicrobial effect of silver particles is mainly due to the release of silver ions, which is often regarded as the main bioactive species [16,17,18]. Besides the silver concentration, the antimicrobial efficacy also depends on the size and shape of the silver particles. Smaller particles have a higher surface-to-volume ratio, which may directly affect the solubility, Ag^+^ release, and thus biological activity [19,20]. Additionally, it was discovered that triangular particles had a higher antibacterial activity than spherical particles due to the presence of more reactive facets [21,22,23].

Despite the excellent antimicrobial properties of silver, questions have arisen about its release into the environment and possible toxic effects [2,16]. Scientific research has shown that silver can enter the human body through skin contact, inhalation, and direct ingestion [5,24]. Skin contact with AgNP-containing dressings led to an elevated silver concentration in the blood, resulting in argyria—a blue or gray discoloration of the skin [25,26]. Moreover, silver particles that enter via the respiratory system can be cytotoxic to alveolar macrophage cells, as well as to epithelial lung cells [27,28]. Directly ingested silver reaches the gastrointestinal tract and accumulates in the liver, resulting in tissue damage [29,30]. Silver can also exert an effect on the environment. When using silver-containing products, silver can end up in the environment and accumulate, for example, in water or soil [2]. Benn et al. revealed that both silver particles and silver ions can easily leak into waste water during the washing of silver-containing textiles [31]. Consequently, microbial-activated sludge systems in waste water treatment can be inhibited, leading to a reduction of water purification [31,32]. Moreover, when silver ends up in the soil, it affects the beneficial bacteria in the soil, which are essential for farming activities [33]. For a more complete overview of the state-of-the-art of human and environmental toxicity studies, we refer to some recently published reviews [2,5,34,35].

Despite the fact that a lot of research is focused on the possible human and ecological risk of silver upon release, it is also critical to take a closer look at the different commercially available silver applications that are responsible for this release [35]. Products that contain ionic silver, colloidal silver, or AgNPs are difficult to trace since they are marketed under numerous brand names and the online availability is enormous. Moreover, reviewing the existing legislation of both EU and non-EU countries shows that only limited specific legislations of silver (nano)particles are available and, with a few exceptions, current labeling regulations do not specifically require the listing of ‘nanomaterial’ as a constituent. This means that consumers can easily buy silver-containing products without knowing which silver form is present [35,36,37,38]. The evolution of the number of silver-containing products occurs more rapidly than the risk assessment. To determine the level of exposure, more information is needed on the concentration of silver, the size of silver, and the form in which silver is present in these products [35]. In view of this knowledge gap, several research groups recently characterized silver-containing products. The presence of silver particles was confirmed in a selection of spray disinfectant products [39], products that are used by or near children [40], and personal care products [41]. Verleysen et al. even proved that AgNPs were released from silver-colored pastry decoration [42]. Our study aims to further investigate this knowledge gap. The presence of silver within different commercially available products that are advertised as containing a certain solid form of silver were analyzed by combining visual analyses, UV-VIS spectroscopy, inductive coupled plasma optical emission spectrometry (ICP-OES), scanning transmission electron microscopy with energy dispersive x-ray spectroscopy (STEM-EDX), and dynamic light scattering (DLS). The obtained results were compared with the label information and the variation between different production batches—which has never been researched before—was investigated.

## 2. Materials and Methods

### 2.1. Products

**Figure 1:**
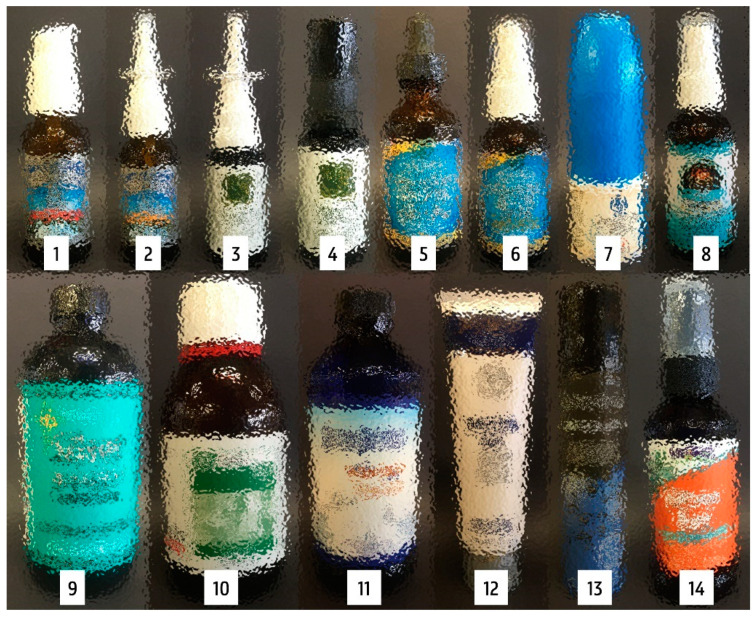
Overview of the 14 different silver products. The label information can be found in Table 1 and Table 2.

Commercially available silver-containing products were selected and purchased from the internet. Fourteen products were ordered from 10 different brands. The different products are shown in Figure 1, and in Table 1 and Table 2, important label information is listed. For products 5, 6, 8, and 9, three different production batches were ordered. All products were advertised as containing a certain solid form of silver, indicated as metallic silver (Ag^0^), colloidal silver, silver hydrosol, or silver (nano)particles, regardless of whether the silver was in combination with soluble ionic silver (Ag^+^). Colloidal silver is a dispersion of metallic silver particles in a liquid. These particles are defined as having a dimension within the size range of 1–1000 nm [11,12,13]. Silver hydrosol is regarded as the purest form of a silver colloid, with water as the liquid phase [43,44]. Nanoparticles are defined as materials where 50% or more of the particles—in an unbound state, as an aggregate or an agglomerate—have one or more external dimensions in the size range of 1–100 nm [14,15].

All of the 14 selected products are intended for human use and are applications of the antimicrobial properties of silver. Most of the products presume to have healing or immune-supporting effects and use a nasal, topical (skin), or oral route of administration. Product 13 is a shoe deo and is used to prevent smelly feet and has to be sprayed in the shoe and not directly on the human body. When analyzing the labels, it was remarkable that—besides the marketing information—a lot of these products mention, in a small font size, the following: ‘*Advantages of this product are neither endorsed nor approved’*, ‘*these statements have not been evaluated by the food and drug administration*’, ‘*this product is not intended to diagnose treat cure or prevent any disease*’, ‘*consult your health care professional before using this product*’, or ‘*this product is not intended for continuous use*’. This indicates the uncertainty of the effects and the caution with which these products must be used. Moreover, the enormous variation in the claimed silver concentration, in combination with the maximum recommended dose, was remarkable.

### 2.2. UV-VIS Spectroscopy and Visual Analysis

Suspensions of silver particles show unique optical UV-VIS absorption spectra and have typical vibrant colors because they have free electrons in the conductivity band. Specific wavelengths of light can drive the conduction electrons in the metal to collectively oscillate, which is a phenomenon known as surface plasmon resonance. These vibrations are specified by the size and shape of the silver particles [45,46]. Therefore, UV-VIS spectroscopy can be used as a characterization technique that provides information about the size and shape of silver particles. Small silver spheres (10–50 nm) typically have a small absorbance peak near a λ_max_ of 400 nm, while larger spheres (100–220 nm) give a broader peak with a λ_max_ that shifts toward longer wavelengths near 500 nm. Moreover, the spectra of larger spheres have a secondary peak at a shorter wavelength, which is a result of quadrupole resonance, in addition to the primary dipole resonance [47,48,49,50]. In addition to the particle size and shape, the concentration of particles has an influence on the observed color and the absorbance at λ_max_. Using a simple dilution, the observed color of particles with the same size and shape can change from, for example, dark orange to bright yellow. Moreover, λ_max_ will not change by dilution, but a decrease in the absorbance at this λ_max_ will be observed [51,52].

UV-VIS spectroscopy was carried out by a Genesys UV-VIS spectrophotometer (Thermo Scientific, Waltham, MA, USA) in Brand® PMMA cuvettes (Merck, Darmstadt, Germany). The absorption spectrum was recorded from 340 to 700 nm. Some products were diluted in Milli Q^®^ (Merck) to measure within the absorbance range of the spectrophotometer. Products 5 and 6 were diluted with a 1:10 ratio and product 7 and 8 with a 1:2 ratio, and a 1:20 dilution of product 9 was measured. Batch 2 of product 8 was not diluted. A visual analysis of the undiluted products was conducted in parallel. As a reference, sodium citrate (NaC)-stabilized AgNPs of 10 and 100 nm were analyzed. The silver concentration of these suspensions was 20 mg L^−1^ and these AgNPs belong to the NanoXact product line of Nanocomposix (San Diego, CA, USA).

### 2.3. Inductive Coupled Plasma Optical Emission Spectrometry (ICP-OES)

The silver concentration was analyzed by the ICP-OES IRIS Intrepid II XSP (Thermo Scientific). Standards for quantification were made out of a 1000 mg L^−1^ Ag^+^ Certipur^®^ stock (Merck). Calibration was conducted between 50 µg L^-1^ to 2 mg L^-1^ Ag^+^. Standards were made in an acid dilutant consisting of Milli Q^®^ with 12% (v/v) of a 37% HCl (Merck) solution and 4% (v/v) of a 65% HNO_3_ (Merck) solution. Additionally, a blank (without Ag^+^) and a control standard of 500 µg L^−1^ Ag^+^ were made. For the sample preparation of the commercially available products, an appropriate amount (Table 3) was diluted in the acid dilutant. After 2 h of digestion at ± 95 °C, the sample was adjusted to a volume of 50.00 mL with the acid dilutant. Every product was analyzed in triplicate. Products that showed turbidity after digestion (products 1, 12, and 13) were filtered with a 589/3 cellulose filter (Whatman, Maidstone, UK) prior to the ICP-OES measurement. Emission was detected at 328 nm and every sample was measured in both axial and radial modus. The average silver concentration of these measurements was reported by the software (TEVA, version 1.6.5., 2001) of the ICP-OES device.

Data analysis was conducted using the R software (R studio 3.6.1). A normal distribution of data was assumed. The assumptions of homoscedasticity for parametric tests were verified by a Levene test. To test if the measured concentrations significantly differed from the concentration on the label, a one-sample T-test was conducted. To test if there were significant differences in concentrations between batches, a one-way ANOVA analysis was used. In case the *p*-value was <0.05, a post-hoc Tukey test was run to see where the differences were situated. The data of the different batches are displayed as boxplots, which provide a graphical view of the median (horizontal line) and quartiles (Q1-Q3, box). The upper whisker is located at the smaller of the maximum concentration values and the Q3 + 1.5 × interquartile range, whereas the lower whisker is located at the larger of the smallest concentration values and the Q1–1.5 × interquartile range.

### 2.4. Scanning Transmission Electron Microscopy with Energy Dispersive X-Ray Spectroscopy (STEM-EDX)

STEM-EDX was conducted at 200 kV with the C_s_-corrected JEM 2200-FS TEM (JEOL, Tokyo, Japan) and a bright-field detector. Holey carbon-coated TEM grids of 200 mesh (Electron Microscopy Sciences, Hatfield, PA, USA) were used. In total, 50 µL of sample was put on the grid or the grid was dipped into the product and air dried before STEM analysis. Products 1 and 12 were treated three times for 15 seconds with the 1020 argon plasma cleaner (Fischione Instruments, Export, PA, USA) prior to STEM analysis, in order to reduce the carbon content from these gels. Chemical information was obtained via the combination of STEM with EDX.

### 2.5. Dynamic Light Scattering (DLS)

DLS was measured by the Zetasizer Nano ZS (Malvern Instruments, Marvern, UK) in backscattering mode at 173° with Zetasizer software 7.11. Product 6 was diluted in Milli Q^®^ (Merck) prior to the DLS measurement with a 1:2 volume ratio. All other products were not diluted. Using the DLS software, silver was selected as the dispersed material. A refraction index (RI) of 0.150 and absorption of 0.001 were entered. Water was selected as the dispersant for all samples. Water had a viscosity of 1.0031 cP and RI of 1.330 at a temperature of 20 °C. Three measurements with an automatic number of runs were completed for each sample.

## 3. Results

### 3.1. UV-VIS Spectroscopy and Visual Analysis

**Figure 2:**
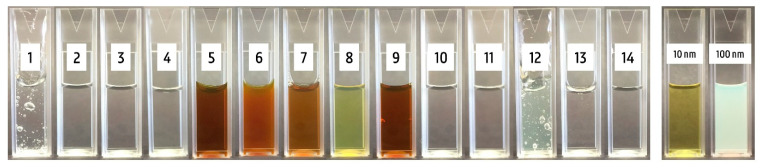
Visual analysis of the 14 different silver products and 10 and 100 nm silver nanoparticles (AgNPs) of Nanocomposix with a concentration of 20 mg L^−1^. Batch 1 is represented for products 5, 6, 8, and 9.

**Figure 3:**
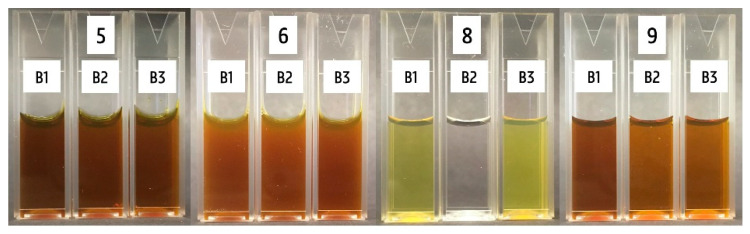
Visual analysis of the three different production batches of products 5, 6, 8, and 9.

Figure 2 shows a visual analysis of the 14 products and the references of 10 and 100 nm silver particle suspensions. The AgNPs of 10 nm had a bright yellow color, while the larger AgNPs of 100 nm were cloudier and had a white-gray color. Product 8 was bright yellow, while products 5, 6, 7, and 9 had a dark orange color, with slide cloudiness in product 6. Product 12 was a gel and displayed a light yellow and more cloudy color. All other products were colorless. The color difference between the different production batches of products 5, 6, 8, and 9 is represented in Figure 3. No batch difference was observed for products 5 and 6, while a remarkable difference was observed for product 8, where batch 2 was colorless. For product 9, batch 1 had a darker orange color compared with the two other batches. 

**Figure 4:**
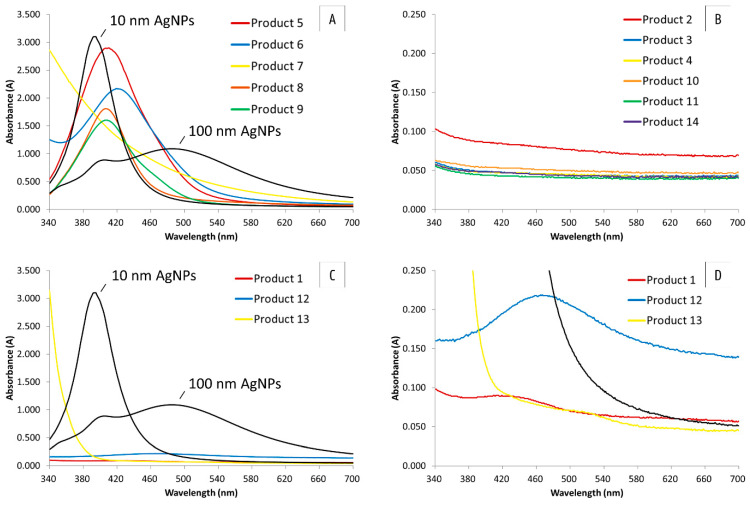
Absorbance spectra of product 5, 6, 7, 8, and 9 (**A**); product 2, 3, 4, 10, 11, and 14 (**B**); and product 1, 12, and 13 (**C,D**) compared with the absorbance spectra of 10 and 100 nm AgNPs of Nanocomposix with a concentration of 20 mg L^-1^. Batch 1 is represented for products 5, 6, 8, and 9. Products 5 and 6 were diluted with a 1:10 ratio and products 7 and 8 with a 1:2 ratio, and a 1:20 dilution of product 9 was measured.

The spectra of the 14 products are represented in Figure 4. AgNPs of 10 nm had a small absorbance peak with a λ_max_ at 394 nm. The larger spheres of 100 nm gave a broader peak with a λ_max_ that shifted towards 485 nm. A secondary peak was observed in this spectrum at around 400 nm. These typical AgNP absorbance spectra were consistent with the literature [47,48,49,50]. The most intense colored products (Figure 4A) exhibited a small absorbance spectrum, with the exception of product 7, where no typical AgNP spectrum was visible and only an increase in absorbance at shorter wavelengths was observed. Batch 1 of products 5, 6, 8, and 9 had a maximum absorbance at 410, 419, 407, and 407 nm, respectively. All of the aforementioned products were diluted prior to UV-VIS spectroscopy, in order to measure within the absorbance range of the spectrophotometer. Products 2, 3, 4, 10, 11, and 14 (Figure 4B) were colorless and only a negligible signal for all tested wavelengths was measured. For the three remaining products—products 1, 12, and 13 (Figure 4C,D)—a zoom of the spectrum is represented. For products 1 and 12, a small increase in absorbance was observed around 440 and 466 nm, respectively. For product 13, the absorbance increased at shorter wavelengths and a minimal increase in absorbance was observed at around 510 nm.

**Figure 5:**
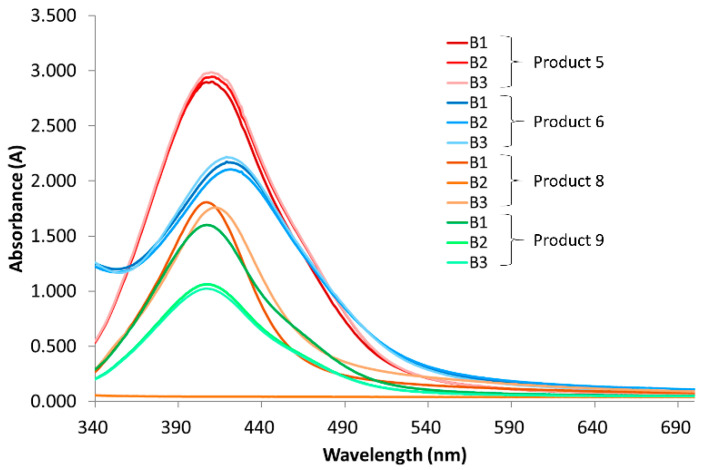
Absorbance spectra of the three different production batches of products 5, 6, 8, and 9. Products 5 and 6 were diluted with a 1:10 ratio and product 8 with a 1:2 ratio, and a 1:20 dilution of product 9 was measured. Batch 2 of product 8 was not diluted.

The spectra of the different production batches of products 5, 6, 8, and 9 are represented in Figure 5. No difference is observed for batches 1, 2, and 3 of products 5 and 6. Concerning product 8, batch 2 displayed a negligible signal for all tested wavelengths, which was consistent with the visual observation, because batch 2 was colorless. The spectra of batches 1 and 3 were similar, with only a small increase in the wavelength of λ_max_ from 407 to 412 nm. The absorbance maximum of batch 1 of product 9 was higher compared with batches 2 and 3. However, the wavelength of this maximum remained unchanged at 407 nm. This analysis corroborated that the color of the first batch of product 9 was more intense than the other ones.

### 3.2. ICP-OES

The silver concentrations of all products were analyzed by ICP-OES and are represented in Table 4. The average and standard deviation of the triplicate analysis, the claimed silver concentration on the label, and the result of a one-sample T-test to test if there is a significant difference between these two are given. The *p*-values can be found in Table S1 of the Supplementary Information (SI) section. It can be seen that for most of the products, the concentration on the label significantly differed from the measured concentration (*p*-value < 0.05). For products 2, 5, 6, 12, and 14, the measured concentration was significantly higher than that on the label, whereas for products 3, 4, and 7, the measured concentration was significantly lower. For product 7, there was almost no silver measured. Moreover, the concentration in product 12 was expressed as mg kg^-1^ because of the difficulty of pipetting the gel. Notwithstanding, the concentration in mg L^-1^ would be even higher because the density is higher than 1 kg L^−1^. For product 8, two out of three batches had a significantly higher measured concentration, whereas for product 9, two batches had a significantly lower concentration and one was significantly higher compared to the label. For product 11, there was no significant difference with the label value and for products 1, 10, and 13, there was no information regarding the silver concentration on the label. Again, it was remarkable that, for product 10, no silver was measured.

**Figure 6:**
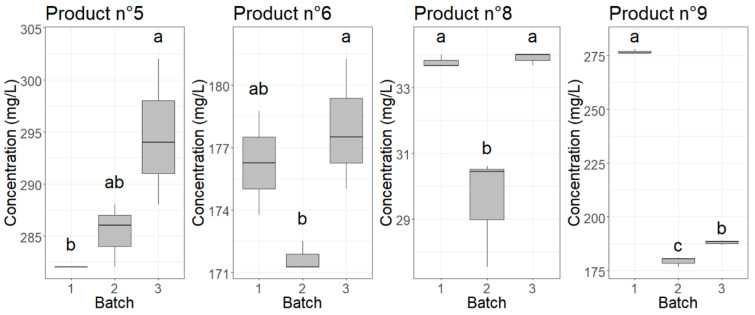
Boxplots showing the variation in measured concentration for the products from which different batches were analyzed. Different letters above the boxes point to significant differences (*p*-value < 0.05) between batches according to a Tukey test.

To test if there was a significant effect of the batch on the measured concentration of products 5, 6, 8, and 9, one-way ANOVA tests were used. The *p*-values can be found in Table S2 (SI section). All *p*-values were lower than 0.05, indicating that at a significance level of α = 0.05, there were significant differences between batches for products 5, 6, 7, and 9. The results of the Tukey tests are illustrated in Figure 6, where different letters point to significant differences between the batches. The percentage difference between the batch with the highest and lowest silver concentration was 4.4%, 3.6%, 13.8%, and 42.7% for products 5, 6, 8, and 9, respectively.

### 3.3. STEM-EDX

**Figure 7:**
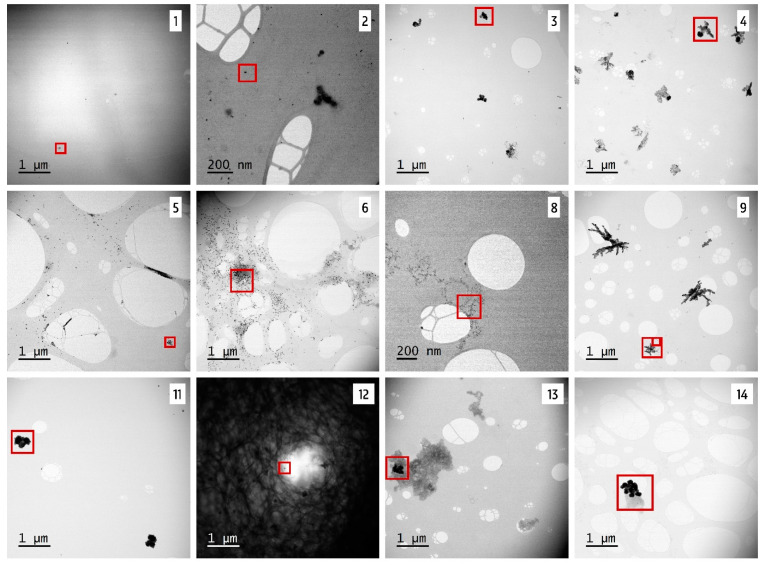
Representative Scanning transmission electron microscopy (STEM) images of 12 different silver products. Batch 1 is represented for products 5, 6, 8, and 9. Zoom and energy dispersive x-ray spectroscopy (EDX) of the red square can be found in Figure 8 and Figure 9.

To verify the presence of silver particles in the consumer products, STEM-EDX analyses were carried out. Products 7 and 10 were not analyzed by STEM-EDX, because no silver concentration was measured by ICP-OES in these samples. Representative STEM images (Figure 7) show that the analyzed products seemed to fall into two categories. The first category included products with a great number of small particles with a spherical shape, such as products 5, 6, and 8. However, most of the products fell into the second category, which included particles with different sizes and irregular and more capricious shapes. This was observed for products 2, 3, 4, 11, 13, and 14. The same observations apply to products 1 and 12, but these two products showed, even after plasma cleaning to reduce the carbon content, more background, possibly because of the gel structure. Product 9 exhibited a combination of the above mentioned categories: Small particles with a spherical shape were observed, surrounded by a few larger and irregular-shaped particles.

**Figure 8:**
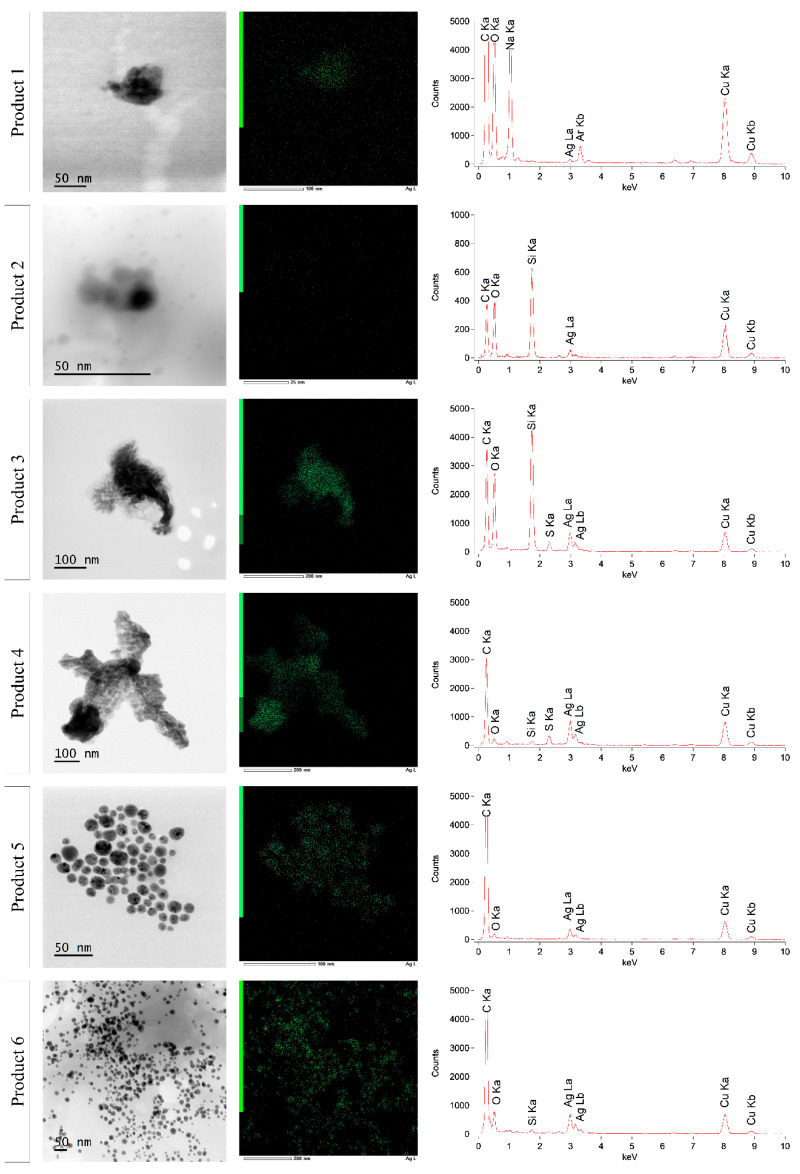
STEM-EDX results of products 1, 2, 3, 4, 5, and 6. Batch 1 is represented for products 5 and 6.

**Figure 9:**
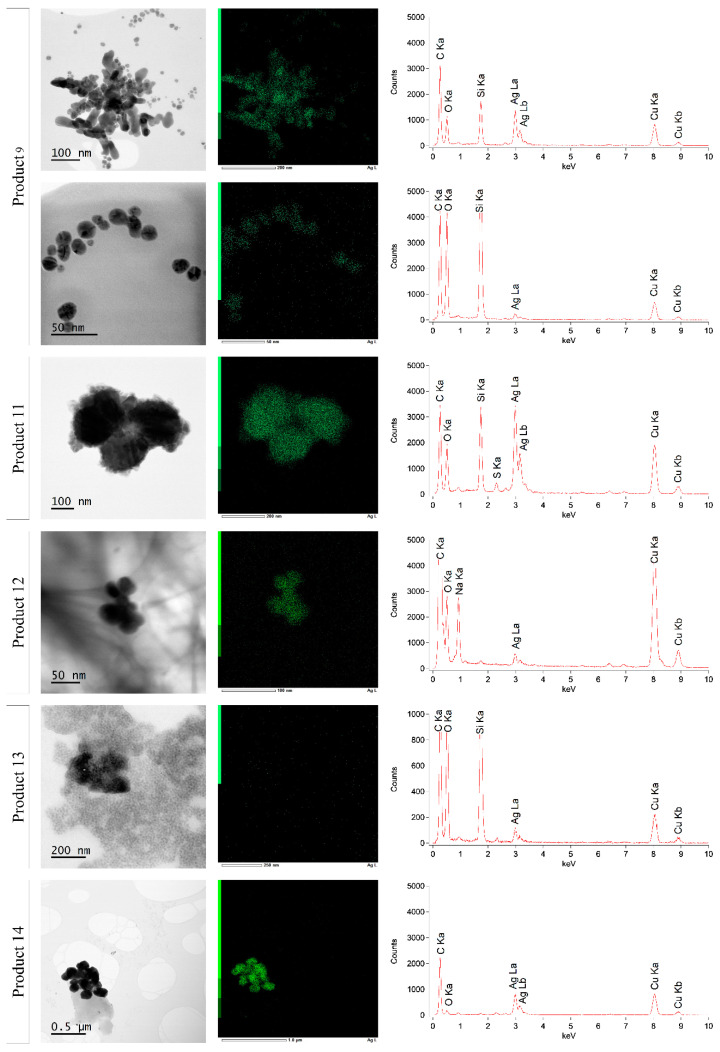
STEM-EDX results of products 9, 11, 12, 13, and 14. Batch 1 is represented for product 9.

Figure 8 and Figure 9 show the STEM-EDX results of products 1–6 and products 9 and 11–14, respectively. The characteristic X-rays were measured over a range of 0 to 20.470 keV. Because only background counts were detected from 10 keV, data of 0 to 10 keV are shown. For all products, carbon (C Ka–0.277 kV) and copper (Cu Ka–8.040 kV and Cu Kb–8.904 kV) were detected. These signals were derived from the used carbon-coated copper grid. Beside this, the presence of oxygen (O Ka–0.525 kV) was measured in all products. This oxygen is at least partly due to the insertion of the specimen holder in the STEM, leading to an interference of ambient air in the vacuum of the STEM. In all analyzed products, the presence of silver was confirmed by the Ag La (2.984 kV) (and Ag Lb (3.150 kV)) peak in the spectrum. The location of the green dots of silver in Figure 8 and Figure 9 indicated that the silver was present within the particle structure, with the exception of products 2 and 13, where the silver counts were much lower and the presence of silver within the particle could not be confirmed. Additionally, sodium (Na Ka–1.041 kV) and sulfur (S Ka–2.307 kV) were detected in products 1 and 12 and in products 3, 4, and 11, respectively. Moreover, the argon plasma cleaning led to the presence of an argon (Ar Kb–3.190 kV) peak in product 1. It was remarkable that silicon (Si Ka–1.739 kV) was apparently detected in most analyzed samples, with the exception of products 1, 5, 12, and 14, where no Si Ka peak was measured.

**Figure 10:**
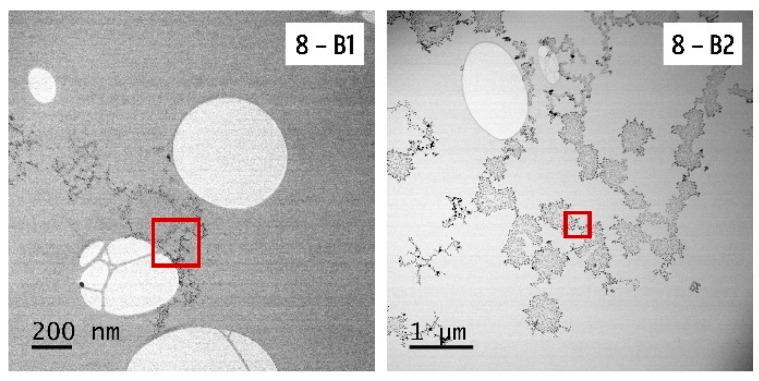
Representative STEM images of batch 1 and 2 of product 8. Zoom and EDX of the red square can be found in Figure 11.

Concerning product 8, batch 1 and 2 were analyzed by STEM-EDX because of the remarkable color difference. Representative STEM images of a colored batch (batch 1) and uncolored batch (batch 2) can be found in Figure 10.

**Figure 11:**
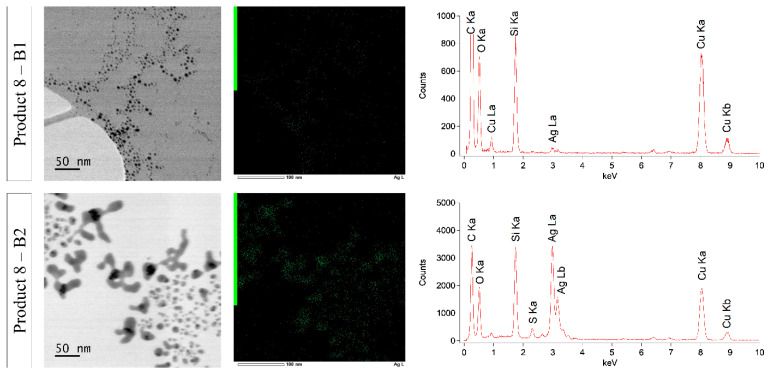
STEM-EDX results of batches 1 and 2 of product 8.

A zoom and EDX analysis of a selected region of these batches is shown in Figure 11. The particles looked different: The ones visible in batch 1 were spherical and small with a small particle size distribution, while the particles in batch 2 had a more irregular and capricious shape and a broader particle size distribution. Similar to all other products, carbon (C Ka–0.277 kV), copper (Cu Ka–8.040 kV and Cu Kb–8.904 kV), and oxygen (O Ka–0.525 kV) were detected by EDX. Beside these elements, silicon (Si Ka–1.739 kV) and silver (Ag La–2.984 kV) were found in both batches of product 8. Despite the low silver counts in batch 1, the presence of silver within the particle structures was confirmed by EDX.

### 3.4. DLS

Finally, all three batches of products 5, 6, 8, and 9 were substantiated through DLS measurements. Only these products were analyzed because the previously reported results showed a great number of spherically-shaped silver particles within these products. For the other products, no silver concentration was detected (products 7 and 10) or a higher polydispersity was observed within the sizes and shapes of the silver particles (products 1–4 and 11–14), meaning that these products would have been less reliable for DLS analysis.

The polydispersity index (PDI), Z-average, and size distribution graph of each measurement are reported in this paper. The PDI is a value that ranges from 0 to 1. It is used to describe the width of the particle size distribution and gives information about the polydispersity of the sample. A PDI value that is higher than 0.400 indicates a polydisperse system. Samples with a high polydispersity may not be suitable for a DLS measurement and the provided data may than be unreliable [53,54,55]. The Z-average is the intensity weighted mean hydrodynamic size of the particles [53,56]. It is important to mention that a hydrodynamic diameter includes the particle’s diameter plus the molecule layer attached or absorbed on the surface, which can lead to an overestimation of the real particle radius [57,58].

**Figure 12:**
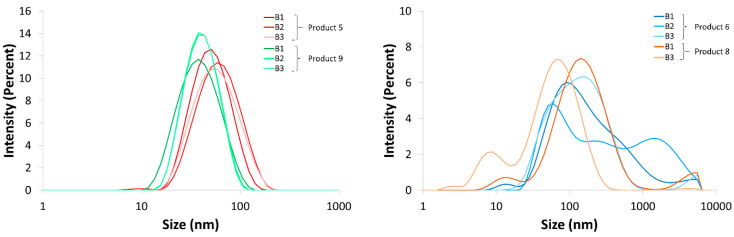
Intensity size distribution of the three different batches of products 5 and 9 (left) and products 6 and 8 (right), excluding batch 2 of product 8, which is represented in Figure S1 (SI section).

The PDI and Z-average of each measurement are listed in Table 5. Figure 12 and Figure S1 (SI section) show the size distribution graphs which represent the intensity percentage as a function of the hydrodynamic size (nm) of the different batches of products 5, 6, 8, and 9.

The PDIs of all batches of products 5 and 9 were smaller than 0.400 (Table 5), indicating a low polydispersity. This was confirmed by the size distribution graphs (Figure 12), which represented a unimodal distribution for the particle sizes. Moreover, no remarkable differences between the batches were observed. The average size of the particles present in products 5 and 9 was around 48 and 34 nm, respectively.

The PDI values of each batch of products 6 and 8 were larger than 0.400 (Table 5). Consequently, polymodal size distribution graphs were reported due to the presence of a large diversity of different particle sizes and thus a highly polydisperse sample. Because of this polydispersity, the interpretation of the Z-average seems incorrect to us because the size of a polymodal distribution graph cannot be defined by just one value. Regarding the size distribution graphs in Figure 12, it can be observed that beside the particles larger than 100 nm, a large number of particles between 10 and 100 nm were present in products 6 and 8. Regarding batch 2 of product 8, the DLS software indicated a poor quality of these measurements, possibly due to a low particle concentration or very small particles, which makes these results impossible to be interpret. Note that this sample had the highest PDI and was the only DLS-measured uncolored sample. The intensity size distribution graph of this sample is represented in Figure S1 (SI section).

## 4. Discussion

The labels of all the characterized products contained the information that solid silver was present in the form of colloidal silver, silver hydrosol, or AgNPs. This indicated that the silver particles should have at least one dimension within a size range of 1 to 1000 nm [11,12,13,14,15]. Through the use of four different techniques (UV-VIS spectroscopy, ICP-OES, STEM-EDX, and DLS), a comparison of the product label information and the obtained results was conducted. Additionally, products among themselves were compared and the variation between three production batches of four different products was examined—something that has rarely been investigated, up until now. Considering all of the obtained results, the different products could be grouped.

The first group contained products 5, 6, 8, and 9. Within these products, a high amount of small, spherically-shaped AgNPs was measured. This was corroborated by UV-VIS spectroscopy, STEM-EDX, and DLS. UV-VIS spectroscopy showed a typical spectrum with a small absorbance peak comparable with the 10 nm AgNPs standard [45,46]. The absorbance spectra maxima of these products were situated around 407–419 nm, corresponding to an estimated particle size of 25-35 nm [48]. DLS measured a Z-average of around 48 and 34 nm for products 5 and 9, respectively. It is important to mention that DLS measures a hydrodynamic diameter, which includes the particle’s diameter plus the molecule layer attached to or absorbed on the surface, what can lead to an overestimation of the real particle radius [57,58]. Products 6 and 8 had a broader size distribution and higher polydispersity compared to products 5 and 9, according to the DLS results. On the other hand, STEM images showed a more polydisperse size distribution in product 9 because larger and irregularly-shaped silver particles were also visible. STEM-EDX of products 5, 6, 8, and 9 confirmed that the observed particles consisted of silver, but also that silicon was detected in three out of four products. This was possibly due to the presence of silica (SiO_2_), which is a molecule that is often used to enhance the colloidal stability of AgNPs [59,60]. The use of silica could also further explain the oxygen peak in the EDX-spectrum. The obtained results confirmed the presence of colloidal silver, as indicated on the product labels. In addition, the labels of products 5 and 6—both from the same manufacturer—described that the highest percentage of AgNPs had a size of 2 nm, which was not confirmed by our data. ICP-OES verified the presence of silver in products 5, 6, 8, and 9, but the measured concentrations were significantly different from the ones on the label. The presence of silicon was also not listed in the ingredient list. 

The labels of products 7 and 10 were advertised as containing nano silver ions or colloidal silver, respectively, but this was not confirmed by our results. Within these products, no silver was detected with ICP-OES, although the product label of product 7 claimed a high silver concentration of 250 mg L^-1^. Moreover, it was misleading that product 7 had an intense orange color because this can be a first indication of AgNP suspensions, but no typical AgNP spectrum was observed by UV-VIS spectroscopy [45,46].

For products 2, 3, 4, 11, and 14, STEM images showed the presence of particles. EDX confirmed that these particles contained silver for almost all products, with the exception of product 2. The observed particles were irregularly shaped and had a widely varying size. Silicon was detected in almost all products, possibly due to the use of SiO_2_ as a stabilizer, as previously described [59,60]. In products 3, 4, and 11, sulfur was also detected. All products were colorless and showed no typical AgNP spectra. The presence of solid silver was confirmed in products 3, 4, 11, and 14, in accordance with the product labels. The particles could not be classified as nano silver-particles as the labels on products 11 and 14 mentioned, because the particles were larger than 100 nm [14,15]. Moreover, the label of product 3 indicated the presence of ultra-small particles of pure zinc, which was not confirmed by STEM-EDX. Silica was not in the ingredient list and the ICP-OES results showed that the claimed silver concentrations significantly differed from the measured concentrations for all products, excluding product 11.

Finally, products 1, 12, and 13 should be discussed. ICP-OES measured silver in all three products, but for product 12, this concentration was significantly different from the label concentration. Despite the high amount of background on the STEM images, possibly because of the carbomer thickeners, EDX confirmed the presence of AgNPs and sodium in products 1 and 12 [61]. In accordance with these results, the UV-VIS spectra of products 1 and 12 demonstrated a small increase in absorbance at around 440 and 466 nm, corresponding to AgNPs of approximately 60 and 80 nm, respectively [48]. The presence of silver hydrosol or nano-silver—as mentioned on the labels of products 1 and 12—was thus confirmed by our results. For product 13, no AgNPs or silica nanoparticles could be detected by STEM-EDX, so the label information was not confirmed by our results. However, the presence of silver was confirmed by both EDX and ICP-OES and silicon was detected by EDX.

Three different production batches of products 5, 6, 8, and 9 were compared. Visual analysis, UV-VIS spectroscopy, and ICP-OES confirmed that batch 2 of product 8 and batch 1 of product 9 differed from the others. A significantly higher silver concentration was measured in batch 1 of product 9. Moreover, a darker color and a higher absorbance spectrum were detected in this product, which is directly related to a higher AgNP concentration [47,62]. Batch 2 of product 8 was even more remarkable: The product looked colorless, only background was detected with UV-VIS spectroscopy, and ICP-OES measured a significantly lower concentration compared to the other batches. The DLS measurement of batch 2 was unreliable due to its poor quality. Notwithstanding, STEM-EDX confirmed the presence of silver particles in batch 2, but the particle structure was totally different compared with a colored batch. The label information of batch 2 of product 8 mentioned ‘*new look, same strength’* and the recommended dose was increased from 1.2 to 3.6 mL daily. Moreover, the ingredient list was changed compared with the other batches. EDTA, often used as a stabilizing agent for AgNPs, was on the ingredient list of batches 1 and 3, but absent on batch 2 [63,64]. This could at least partly explain why the particles in batch 2 had a more irregular and capricious shape due to poorer stabilization. The particle characteristics, particle concentration, and silver concentration differed for the production batches of products 8 and 9. The lowest and highest concentrated batch had a difference of 13.8% and even 42.7% for products 8 and 9, respectively. This was similar for products 5 and 6, where a significant difference in silver concentration was measured by ICP-OES for the different batches of these products, with a percentage difference of 4.4% and 3.6%, respectively. 

In four out of the 14 products, a high amount of spherically-shaped AgNPs was detected, while the silver particles in other products had an irregular shape and a higher size polydispersity. On the other hand, no (solid) silver was detected in four other products. A significant difference between the claimed and measured silver concentration was noticed for almost all products and the concentration varied between approximately 2 and 300 mg L^−1^. Finally, it was remarkable that a high degree of variability between production batches of the same product was observed, regarding both particle characteristics and the silver concentration.

## 5. Conclusion

Due to the beneficial antimicrobial properties of silver particles, the number of commercially available applications of silver-containing products keeps growing. The free availability and widespread use of the products result in human and environmental exposure and consequently, in an increase of silver particle toxicity studies. Because the particle size, shape, and concentration all have an influence on the toxicity, a more complete understanding of these characteristics in commercially available products is crucial for determining the risk involved with human and environmental exposure.

To the best of our knowledge, this is the first report to analyze a wide collection of different silver-containing products and different production batches by the use of four techniques: UV-VIS spectroscopy, ICP-OES, STEM-EDX, and DLS. Our results demonstrated a high variability in silver particle characteristics, silver concentrations, and production batches. A significant difference between the claimed and measured silver concentration was observed for almost all products. Although more data are needed, it is clear from the presented results that manufacturers produce and label silver-containing products with a certain imprudence.

Because of concerns regarding the increased exposure and toxicity of silver, we advise that a more rigorous approach is needed in the manufacturing, labeling, and consumer use of silver-containing products. We want to encourage competent government authorities to take our results into account and handle the imprudence in producing and labeling silver-containing products. The limited specific legislation is at least partly responsible for our observations. We encourage the need for increased cooperation between the government and the scientific world to assess this problem. The general idea of ‘no data, no matter’ can be dangerous for our society and urgently needs to be addressed.

## Figures and Tables

**Table 1:** Label information of the 14 different silver products (part 1).

Product	Brand	Product Type	Description Silver Form	Claimed Ag Concentration (mg L^−1^)	Maximum Recommended Daily Dose (mL)
1	A	Skin gel	Silver hydrosol, metallic silver	Unknown	Unknown
2	A	Nasal spray	Silver hydrosol, silver ions, silver nanoclusters (0.8 nm)	10	7
3	B	Nasal spray	Colloidal silver, ultra-small particles pure silver	15	0.5
4	B	Skin spray	Colloidal silver, ultra-small particles pure silver	15	0.5
5	C	Oral dietary supplement (dropper)	Colloidal silver, silver particles (highest percentage of 2 nm)	250	5
6	C	Oral dietary supplement (spray)	Colloidal silver, silver particles (highest percentage of 2 nm)	150	1.5
7	D	Nasal spray	Nano silver ion	250	0.3
8	E	Oral dietary supplement (spray)	Colloidal silver, silver with minute particle size	30	1.2 or 3.6
9	F	Oral dietary supplement (liquid)	Colloidal silver	200	30
10	G	Oral dietary supplement (liquid)	Colloidal silver	Unknown	Unknown
11	H	Oral dietary supplement (liquid)	Silver sol technology (metallic nano-silver particle with thin multivalent Ag_4_O_4_ coating)	10	15
12	H	Skin gel	Silver sol technology, nano-silver	20	Unknown
13	E	Shoe deo	Silver nanoparticles	Unknown	Unknown
14	F	Oral dietary supplement (spray)	Silver sol technology	10	15

**Table 2:** Label information of the 14 different silver products (part 2).

Product	Batch	Lot/Batch Number	Expiration Date	Country of Origin	Ingredients (Beside Silver)
1	1	GJ018T	07/2021	USA	Carbomer (carbopol), sodium hydroxide
2	1	GK104S	09/2021	USA	Pharmaceutical-grade purified water
3	1	NZS124102018	10/2021	The Netherlands	Ultra-small particles pure zinc, purified water
4	1	NS103102018	10/2021	The Netherlands	Purified water
5	1	7103	05/2022	USA	Pharmaceutical-grade deionized water
	2	7605	10/2022		
	3	7622	10/2022		
6	1	7113	05/2022	USA	Organic echinacea, oregano leaf tincture, aloe vera leaf juice, deionized water, licorice extract
	2	7323	07/2022		
	3	7634	10/2022		
7	1	20180502	01/05/2020	China	*Chrysanthemi indici* flos, *Angelicae dahuricae* radix, centipedae herba, xanthii fructus, prunellae spica, propolis, borneolum syntheticum, polyhexamethylene biguanide
8	1	FG-90226, REV G172	08/2020	USA	Potassium alginate, distilled water, ethylenediaminetraacetic acid (EDTA)
	2	FG-97614, REV D184-A	09/2020		Distilled water
	3	FG-87919, REV G171	06/2020		Potassium alginate, distilled water, EDTA
9	1	10719A	04/2022	USA	Water
	2	30119A	10/2022		
	3	Unknown	Unknown		
10	1	P02745	06/2022	The Netherlands	Purified water
11	1	18052	02/2021	USA	Deionized water
12	1	18263	09/2021	USA	Deionized water, tetraethylammonium, carbomer
13	1	NDS DW.04.03.2021	04/03/2021	Poland	Silica nanoparticles, aliphatic hydrocarbons, benzyl salicylate, citronellol, hexyl cinnamal, butylphenyl methylpropional
14	1	19036	02/2022	USA	Deionized water

**Table 3:** Product amount used for inductive coupled plasma optical emission spectrometry (ICP-OES) sample preparation in a final volume of 50.00 mL.

Product	Amount in 50.00 mL
1	1.50 g
2	3.00 mL
3	5.00 mL
4	2.50 mL
5	0.25 mL
6	0.40 mL
7	5.00 mL
8	1.50 mL
9	0.25 mL
10	20.00 mL
11	5.00 mL
12	0.75 g
13	1.50 g
14	0.75 mL

**Table 4:** ICP-OES results of the 14 different silver products and the three different production batches of products 5, 6, 8, and 9. One-sample T-tests were used to test if the measured concentration was significantly different from the claimed concentration on the label.

Product	Batch	Ag^+^ Conc. ± SD(N = 3)	Claimed Ag Conc. (mg L^–1^)	Is Difference Significant (*p*-value < 0.05)?
1	1	26.22 ± 0.20 mg kg ^−1^	Unknown	-
2	1	12.22 ± 0.01 mg L^−1^	10	Yes
3	1	2.13 ± 0.03 mg L^−1^	15	Yes
4	1	6.11 ± 0.84 mg L^−1^	15	Yes
5	1	282.00 ± 0.00 mg L^−1^	250	Yes
	2	285.33 ± 3.06 mg L^−1^		Yes
	3	294.67 ± 7.02 mg L^−1^		Yes
6	1	176.25 ± 2.50 mg L^−1^	150	Yes
	2	171.67 ± 0.72 mg L^−1^		Yes
	3	177.92 ± 3.15 mg L^−1^		Yes
7	1	0.01 ± 0.01 mg L^−1^	250	Yes
8	1	33.78 ± 0.19 mg L^−1^	30	Yes
	2	29.52 ± 1.72 mg L^−1^		No
	3	33.89 ± 0.19 mg L^−1^		Yes
9	1	276.67 ± 1.15 mg L^−1^	200	Yes
	2	179.27 ± 2.32 mg L^−1^		Yes
	3	187.93 ± 1.17 mg L^−1^		Yes
10	1	0.00 ± 0.00 mg L^−1^	Unknown	-
11	1	10.54 ± 1.33 mg L^−1^	10	No
12	1	22.27 ± 0.46 mg kg^−1^	20	Yes
13	1	2.83 ± 0.30 mg kg^−1^	Unknown	-
14	1	11.40 ± 0.12 mg L^−1^	10	Yes

**Table 5:** Polydispersity index (PDI) and Z-average of the three different batches of products 5, 6, 8, and 9. PDI values > 0.400 are indicated in grayscale.

Product	Batch	PDI	Z-Average (nm)
5	1	0.204	43.38
	2	0.217	50.97
	3	0.222	48.73
6	1	0.455	114.3
	2	0.788	126.8
	3	0.426	99.83
8	1	0.556	100.9
	2	0.853	1879
	3	0.568	34.46
9	1	0.238	31.86
	2	0.207	34.83
	3	0.216	34.14

## Supplementary Materials

The following are available online at https://www.mdpi.com/2079-4991/10/7/1394/s1: Figure S1. Intensity size distribution of batch 2 of product 8; Table S1. *P*-values of one-sample T-tests to test if the measured concentration was significantly different from the concentration on the label; Table S2. *P*-values of one-way ANOVA tests to test if there was a significant effect of the batch on the measured concentration.
