# Supplementary material for: High Variability in Silver Particle Characteristics, Silver Concentrations, and Production Batches of Commercially Available Products Indicates the Need for a More Rigorous Approach
Source: Nanomaterials (Basel). 2020 Jul 17;10(7):1394. doi: 10.3390/nano10071394 (PMC7408040; doi:10.3390/nano10071394)
Supplement: Supplementary file 1 [file nanomaterials-10-01394-s001.pdf]

Supplementary Information section

High Variability in Silver Particle Characteristics, Silver Concentrations, and Production Batches of Commercially Available Products Indicates the Need for a More Rigorous Approach

Ilse De Leersnyder, Hannes Rijckaert, Leen De Gelder, Isabel Van Driessche and Pieter Vermeir

Supplementary information section

Table S1. P-values of one-sample T-tests to test if the measured concentration was significant different from the concentration on the label.

Product	Batch	P-value (one-sample T-test)
1	1	-
2	1	0.000
3	1	0.000
4	1	0.003
5	1	0.003
	2	0.003
	3	0.008
6	1	0.003
	2	0.004
	3	0.004
7	1	0.000
8	1	0.001
	2	0.679
	3	0.001
9	1	0.000
	2	0.004
	3	0.003
10	1	-
11	1	0.555
12	1	0.013
13	1	-
14	1	0.023

Table S2. P-values of one-way ANOVA tests to test if there was a significant effect of batch on the measured concentration.

Product	P-value (one-way ANOVA)
5	0.030
6	0.042
8	0.003
9	0.000

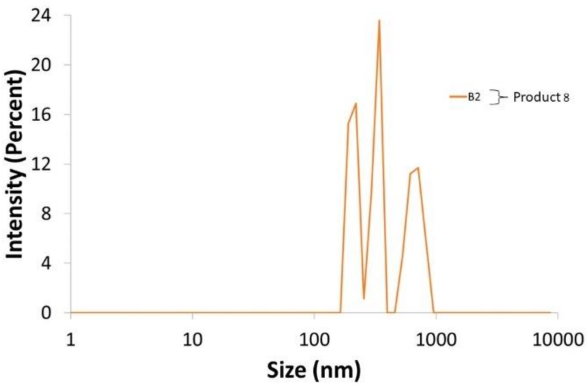

Figure S1. Intensity size distribution of batch 2 of product 8.
